# Polypills for Primary Prevention of Cardiovascular Disease: A Systematic Review and Meta-Analysis

**DOI:** 10.3389/fcvm.2022.880054

**Published:** 2022-04-14

**Authors:** Omneya A. Kandil, Karam R. Motawea, Merna M. Aboelenein, Jaffer Shah

**Affiliations:** ^1^Faculty of Medicine, Alexandria University, Alexandria, Egypt; ^2^Medical Research Center, Kateb University, Kabul, Afghanistan

**Keywords:** primary prevention, cardiovascular events, antihypertensives, polypill, lipid-lowering

## Abstract

**Purpose:**

To evaluate the effect of polypills on the primary prevention of cardiovascular (CV) events using data from clinical trials.

**Methods:**

We searched PubMed, Web of Science, EBSCO, and SCOPUS throughout May 2021. Two authors independently screened articles for the fulfillment of inclusion criteria. The RevMan software (version 5.4) was used to calculate the pooled risk ratios (RRs) and mean differences (MDs), along with their associated confidence intervals (95% CI).

**Results:**

Eight trials with a total of 20653 patients were included. There was a significant reduction in the total number of fatal and non-fatal CV events among the polypill group [RR (95% CI) = 0.71 (0.63, 0.80); *P*-value < 0.001]. This reduction was observed in both the intermediate-risk [RR (95% CI) = 0.76 (0.65, 0.89); *P*-value < 0.001] and high-risk [RR (95% CI) = 0.63 (0.52, 0.76); *P*-value < 0.001] groups of patients. Subgroup analysis was performed based on the follow-up duration of each study, and benefits were only evident in the five-year follow-up duration group [RR (95% CI) = 0.70 (0.62, 0.79); *P*-value < 0.001]. Benefits were absent in the one-year-or-less interval group [RR (95% CI) = 0.77 (0.47, 1.29); *P*-value = 0.330]. Additionally, there was a significant reduction in the 10-year predicted cardiovascular risk in the polypill group [MD (95% CI) = −3.74 (−5.96, −1.51); *P*-value < 0.001], as compared to controls.

**Conclusion:**

A polypill regimen decreases the incidence of fatal and non-fatal CV events in patients with intermediate- and high- cardiovascular risk, and therefore may be an effective treatment for these patients.

## Introduction

Globally, the prevalence of cardiovascular disease (CVD) has almost doubled in the last 30 years, with CVD mortality increasing from 12.1 million cases in 1990 to 18.6 million cases in 2019. The leading cause of these numbers include suboptimal preventive methods and uncontrolled atherosclerotic cardiovascular disease (ASCVD) risk factors (1, 2). To combat these issues, the World Heart Federation launched a global campaign to reduce premature mortality due to CVD by 25 percent by 2025 (3). In fact, Atherosclerotic cardiovascular diseases are due to a chronic lifetime exposure of individuals to CVD risk factors such as high blood pressure levels (4). In 2003, Wald and Law coined the term polypill to describe a combination of active pharmaceutical components that had the potential to reduce the burden of cardiovascular diseases by more than 80% (5). The polypill has proven to increase adherence rates for secondary prevention, which is significant because roughly one third of people face adherence issues to blood pressure or lipid lowering medications, and that a large proportion of all CVD events (about 9% in Europe) is due to poor adherence to vascular medications alone (6, 7). However, the use of such a pill in primary prevention – especially for those with an intermediate to high risk of cardiovascular disease – is not well-understood, given that no studies have been performed to assess the long-term effectiveness of such an intervention (8). Intermediate risk is defined is defined by a Framingham score of 10–20% or an INTERHEART score of 10–15, while high risk is defined by a Framingham score of >20% or an INTERHEART score of >16. Despite its cost-effectiveness and ease of use, multiple drawbacks have hampered the wide scale prescription of polypills, most notably physician hesitancy and the inability to tailor doses according to patient needs (9). Trials were designed to investigate the benefits of polypill use, yet they generally assessed the improvement in CVD in terms of risk factors such as blood pressure and lipid profiles rather than clinical outcomes such as fatal and non-fatal cardiovascular events (8). Moreover, these studies were usually performed in developing countries and could not be generalized to developed countries (10). New trials have since been published that account for such problems. Considering the newly published studies, the aim of this meta-analysis is to focus on assessing the effectiveness of polypills in preventing clinical outcomes as strokes and Myocardial infarctions and mortality rather than assessing their effect on lipid or blood pressure changes,such parameters have already been discussed in several previous studies so centered our attention on tangible clinical outcomes instead. We also discussed possible solutions to any drawbacks that this treatment might possess.

## Methods

### Search and Identification of Studies

A comprehensive literature search was carried out in May 2021 on the following databases: PUBMED, WOS, EBSCO, and SCOPUS. Search terms used were (polypill OR “fixed dose” OR “drug combination” OR “drug combinations”) AND (“heart outcomes prevention” OR “primary prevention” OR “Framingham score” OR “estimated 10-year cardiovascular risk”). Reviews and book chapters were excluded. Additional manual searches were performed through the “related articles” feature in PubMed. Lastly, all references from the reviewed articles were checked for any articles that might have been missed in the original literature search.

### Selection Process and Inclusion Criteria

Once the searches were completed, the software programme Covidence was used to perform the de-duplication of citations and for the screening process. From the searches, two review authors (M.A, O.K) reviewed the title and abstract of each paper and retrieved potentially relevant references. Following this initial screening, we obtained the full text of potentially relevant studies, and the two authors (M.A, O.K) independently screened them using predetermined inclusion criteria. The inclusion criteria are as follows: (1) trials that include the use of a fixed-dose combination of at least 2 drugs, one of them being a lipid lowering- and the other being a blood pressure lowering-drug; (2) trials that include (even if not limited to) primary prevention of cardiovascular disease in patients with at least one cardiovascular risk factor or calculated cardiovascular risk score but no previous cardiovascular events; and (3) trials that either report the effect of the FDC on the incidence of cardiovascular events such as stroke or MI or that report the 10-year predicted cardiovascular risk (Framingham Score) after FDC use. Disagreements were resolved by discussion and a decision was reached after agreement between the reviewers. We only included trials in our meta-analysis and excluded any observational studies, case reports, and non-English articles.

### Outcomes

The primary outcome was the effect of Polypill on total fatal and non-fatal cardiovascular events as Myocardial infarction, stroke, heart failure or angina. Secondary outcomes were effect of polypill on the predicted 10-year cardiovascular risk for the studies which lacked data regarding our primary outcome, so the mean difference of the cardiovascular risk score was used as a predictor of these fatal and non-fatal CV events. The number of participants who discontinued treatment due to adverse effects and the total adverse events along with myalgia were also analyzed.

### Data Extraction

Two review authors (O.K, M.A) independently extracted data and consulted the principal investigator when needed. They extracted details of the study design, participant characteristics, study setting, intervention, and comparator. They also extracted outcome data, which included the composite of death from cardiovascular causes and adverse events such as myocardial infarction, stroke, heart failure, resuscitated cardiac arrest, arterial revascularization, and angina. The study quality was assessed as part of the data extraction strategy with RoB 2 revised Cochrane risk-of-bias tool for randomized trials. The tool is structured into a fixed set of domains of bias, focussing on trial's design, conduct, and reporting. In each domain, signaling questions elicit information about features of the trial that are relevant to risk of bias. A judgement about the risk of bias in each domain is generated based on answers to the signaling questions. Judgement can express ‘some concerns', be 'Low' or 'High' risk of bias (11). Quality of evidence was assessed using GRADE approach through tools on their website gradepro.

### Statistical Analysis

A meta-analysis was carried out to evaluate the impact of a fixed-dose combination of lipid lowering and blood pressure lowering drugs, as compared to placebo or non-pharmacological intervention among participants without cardiovascular disease that were at an intermediate or high risk of developing CVD. RevMan 5.4 was used for statistical analysis. If we detected heterogeneity among studies (*p*-value <0.05), we used leave one out test or subgroup analysis to resolve the heterogeneity. We used fixed effects model if we observed no heterogeneity among studies, otherwise a random effects model was used if a significant heterogeneity was observed (*p*-value < 0.05). Our analysis followed the PRISMA Statement Checklist to ensure a high-quality review (12).

## Results

### Study Inclusion

**Figure 1:**
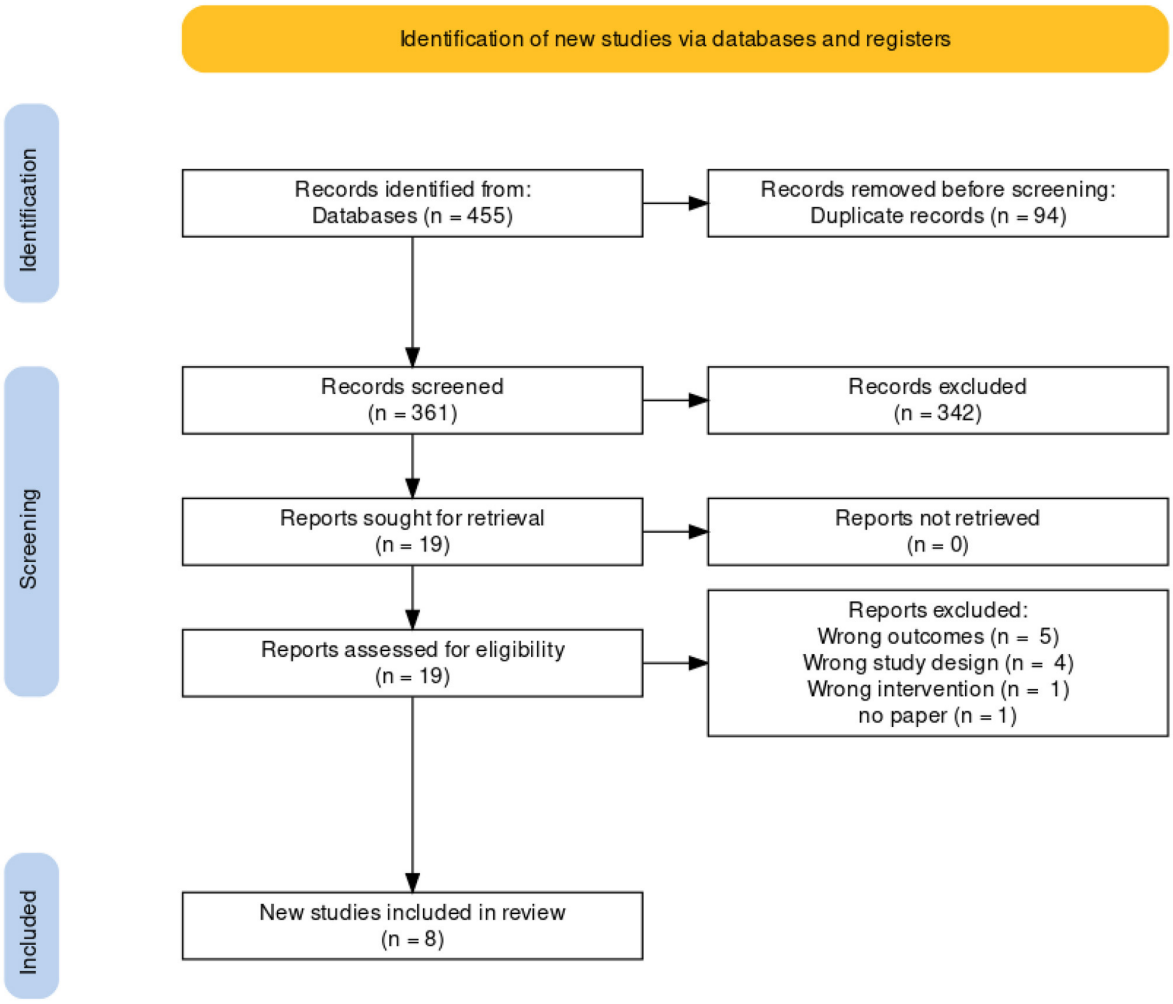
PRISMA flowchart.

**Figure 2:**
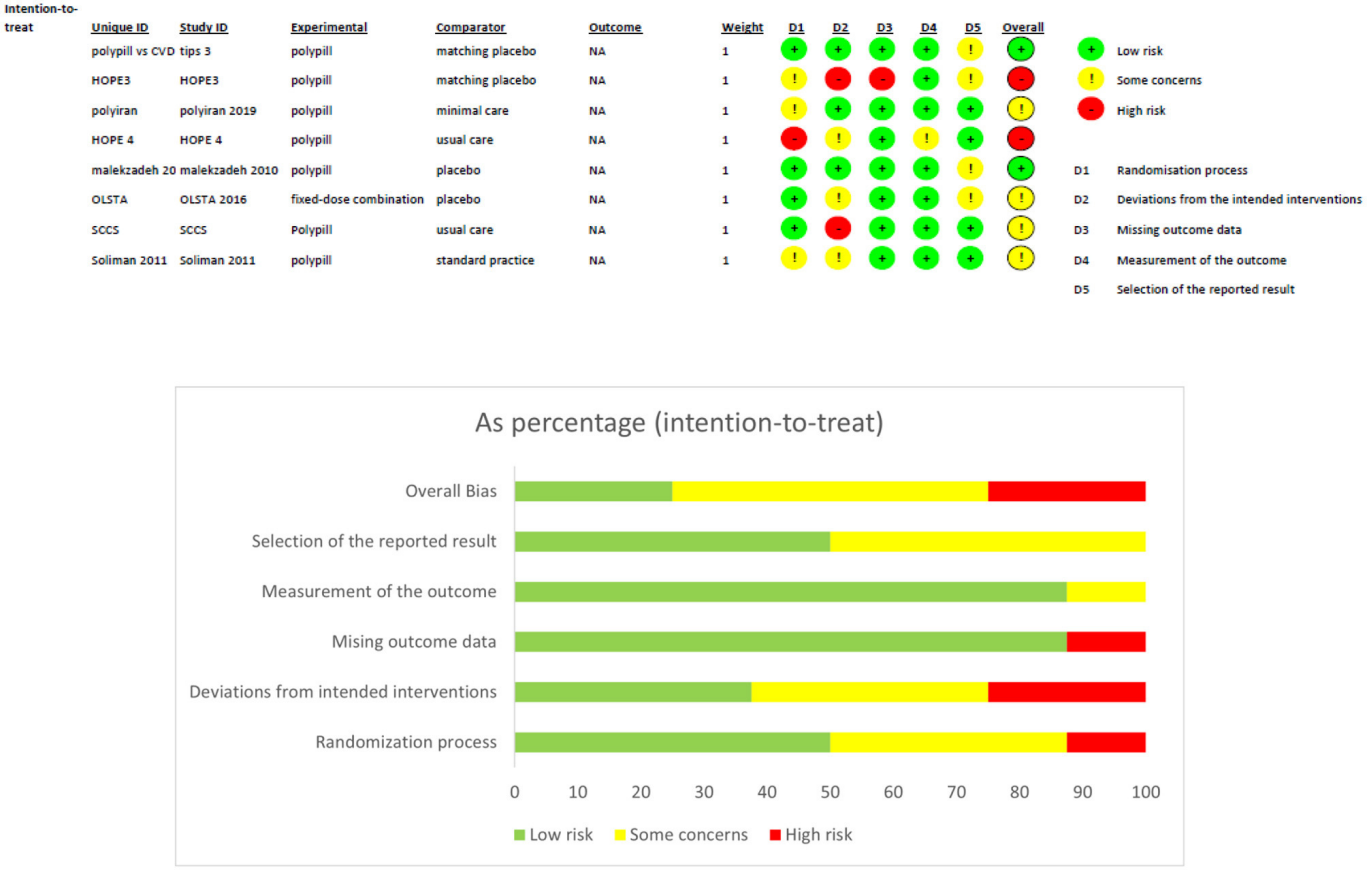
Risk of bias assessment and graph.

Four hundred and fifty five publications were extracted from literature search and, after the removal of duplicates, 361 moved on to the next stage of review. After title and abstract screening, 18 studies were eligible for a full-text review. Of these, eight studies were eligible for inclusion in our meta-analysis. A detailed description is illustrated in the PRISMA flow chart (Figure 1). Of the eight trials included, six contained data that compared the incidence of fatal and non-fatal cardiovascular events – such as stroke, myocardial infarction, heart failure, cardiovascular death, and revascularisation – between patients who received a polypill treatment and those who received placebos/minimal pharmacological care. Two studies did not contain information about CV events, so we instead compared the mean difference in the 10-year predicted cardiovascular risk between patients who received the polypill and patients who received placebos/minimal care. We were able to extract this outcome from a third study as well, so its results were included along with the other two in the Meta-Analysis. The summary and the risk of bias of the included studies are shown in Table 1 and Figure 2, respectively. The quality of evidence table is presented in Table 2.

**Table 1:** Summary of the included studies (13–21).

**ID**	**NCT**	**Study region**	**Design**	**Duration**	**Study arms**	**Endpoints (outcomes) conclusion**	**Conclusion**
TIPS 3 - (16)	NCT01646437	India Philippines Colombia Bangladesh Canada Malaysia Indonesia Tunisia Tanzania	Double-blind, RCT of polypill vs. placebo	4.25 years	1-Experimental: Polycap vs. matching placebo 2- Experimental: Aspirin vs. matching placebo 3- Experimental: Vitamin D vs. matching placebo	Primary: 1- composite of CV events, which includes major CVD (ie, CV death, non-fatal stroke, non-fatal MI) 2- heart failure, resuscitated cardiac arrest, or arterial revascularization. 3- Secondary outcomes are (1) major CVD and (2) the composite ofmajor CVD, heart failure, resuscitated cardiac arrest, arterial revascularization, or angina with evidence of ischemia 3- cancer 4- heart failure, resuscitated cardiac arrest, arterial revascularization, or angina with evidence of ischemia. Other outcomes: 1- all-cause mortality 2- incident and recurrent CV events 3- visual acuity 3- age-related macular degeneration 4- cognitive function 5- adverse events (including bleeding 6- economic analysis related outcomes	Results of the TIP-3 study will be key to determining the appropriateness of FDC therapy as a strategy in the global prevention of CVD
HOPE 3, (26)	NCT00468923	Canada (in Asians)	Quadruple, RCT of polypill vs. placebo	5.6 years	Placebo Comparator: Rosuvastatin Placebo Comparator: Candesartan/HCT	primary: 1- The composite of; Cardiovascular death, non-fatal myocardial infarction, non-fatal stroke. 2- resuscitated cardiac arrest, non-fatal myocardial infarction, heart failure, arterial revascularizations Secondary: 1- Total mortality 2- The components of the co-primary endpoints	Candesartan/ hydrochlorothiazide had fewer effects in reducing blood pressure in Chinese and rosuvastatin reduced low-density lipoprotein cholesterol to a lesser extent in Asians compared with non-Asians. There was no overall reduction in clinical events with lowering blood pressure in either Asians or non-Asians, whereas there were clear and consistent benefits with lipid lowering in both. Despite extensive analyses, there is no obvious explanation for the observed findings. Future studies need to include larger numbers of individuals from different regions of the world to ensure that the results of trials are applicable globally.
Polyiran	NCT01271985	Iran	A two-group, pragmatic, cluster randomized trial of polypill vs. minimal care	5 years	Experimental: polypill and minimal care (polypill arm) no intervention: minimal care	1- primary outcome: occurrence of MCVE within five years of enrolment 2- Secondary outcomes: a- non-cardiovascular causes of death (including neoplastic, respiratory, hepatic, renal and other medical causes), b- adherence to the polypill, c- effects of the interventions on MCVE, d- changes in blood pressure and low-density lipoprotein (LDL) cholesterol during the trial.	Use of a fixed-dose combination of aspirin, atorvastatin, and two blood pressure-lowering drugs was associated with a significantly lower risk of major cardiovascular events in individuals aged 50–75 years in a real-life setting. This pragmatic trial provides evidence that a polypill strategy could be considered as part of preventive programmes to reduce cardiovascular disease burden among eligible adults, especially in LMICs
HOPE 4	NCT01826019	Colombia and Malaysia	Cluster-randomized controlled trial of polypill Vs. usual care	12 months	Experimental: combinations of anti-hypertensive medications (both low and high doses) and a lipid lowering agent (e.g., statin) No intervention: usual care	Primary outcomes: 1- change in Framingham Risk Score (FRS) between the intervention and control after1 year 2- Difference in major CV events at 6 years Secondary outcomes: 1- Change in systolic BP (SBP) 2- Proportion of participants with well-controlled blood pressure at 6 and 12 months 3- Change in HDL, LDL, total cholesterol, triglycerides, and glucose levels 4- Change in smoking status 5- Change in IHRS and ChRS 6- no. of participants recieving anti-hypertensives 7- clinical events (e.g. death, CVD development, hospitalizations)	This strategy is effective, pragmatic, and has the potential to substantially reduce cardiovascular disease compared with current strategies that are typically physician based.
SCCS, (20)	NCT02278471	United States	Open-label, parallel RCT comparing polypill to usual care	12 months	Experimental arm: Polypill No Intervention arm: Usual Care	Primary outcomes: 1- Systolic Blood Pressure 2-Medication Adherence-Percentage of Pills Taken 3-LDL Cholesterol Secondary Outcome Measures: 1-Systolic Blood Pressure 2- Medication Adherence 3- Drug Metabolite Profile 4- Insulin Resistance 5−*Inflammatory* Profile	A polypill-based strategy led to greater reductions in systolic blood pressure and LDL cholesterol level than were observed with usual care in a socioeconomically vulnerable minority population
Soliman, (21)	NCT00567307	Sri Lanka	Open-label, parallel RCT comparing Polypill to Standard Practice	3 months	Experimental: The Red Heart Pill 2b (Polypill) (A) Active Comparator: Standard Practice Group (B)	1- Reduction of the Estimated 10-year Total Cardiovascular Risk Score	Polypill was simpler and achieved comparable risk factor reductions, highlighting its potential usefulness in the prevention of CVD. Further studies assessing the Polypill in developing countries should take into consideration the study design lessons and challenges that we encountered.
Malekzadeh et al. (18)	ISRCTN43076122	Iran	Double- blind, parallel RCT of polypill vs placebo	12 months	Experimental: polypill non- interventional: placebo	Primary outcome measure Combined cardiovascular events (MI, new onset angina, coronary artery surgery, stroke or sudden cardiac death) Secondary outcome measures 1. Total mortality 2. Gastrointestinal bleeding	The effects of the polypill on blood pressure and lipid levels were less than anticipated, raising questions about the reliability of the reported compliance. There is a case for a fully powered trial of a polypill for the prevention of cardiovascular disease.

**Table 2:** Quality of evidence table using GRADE approach.

**Certainty assessment**							**No of patients**		**Effect**		**Certainty**	**Importance**
**No of studies**	**Study design**	**Risk of bias**	**Inconsistency**	**Indirectness**	**Imprecision**	**Other considerations**	**intervention**	**comparison**	**Relative (95% CI)**	**Absolute (95% CI)**		
**Total fatal and non fatal cardiovascular events**												
6	Randomized trials	Not serious	Serious^a^	Not serious	Not serious	None	432/9993 (4.3%)	619/10154 (6.1%)	RR 0.71 (0.63 to 0.80)	18 fewer per 1,000 (from 23 fewer to 12 fewer)	⊕⊕⊕○ Moderate	Critical
**Difference in 10 year predicted cardiovascular risk**												
3	Randomized trials	Serious	Not serious	Serious^a^	Not serious	None	854	951	-	MD 3.74 lower (5.96 lower to 1.51 lower)	⊕⊕⊕○○ Low	Critical
**Total adverse events**												
5	Randomized trials	Not serious	Not serious	Not serious	Not serious	None	541/6457 (8.4%)	408/6387 (6.4%)	RR 1.03 (0.70 to 1.51)	2 more per 1,000 (from 19 fewer to 33 more)	⊕⊕⊕ High	Important

*CI, confidence interval; MD, mean difference; RR, risk ratio*.

a *There are different follow up durations in the studies so by performing subgroup analysis to duration less than 12 months and less than 5 years heterogeneity was resolved. There is also another subgrouping that may account for this heterogeneity which is based on the cardiovascular risk into intermediate and high subgroups so we performed also a subgroup analysis for this and again this resolved the heterogeneity*.

### Analyses

The total number of patients included in the polypill treatment group of our meta-analysis is 10,240, with a mean age of 61.1. The placebo/minimal care group included 10,413 patients, with a mean age of 61.38. Females represented 43.7% of the total study population, and males represented 56.4%. Detailed baseline characteristics are shown in Table 3.

**Table 3:** Baseline characteristics of patients (13–21).

	**Study arms**		**Number of patients in each group**		**Age (years)**			
**ID**	**Intervention (the name and the form of the durg)**	**Control (placeob or another durg)**	**Intervention**	**Control**	**Intervention**	**Control**	**Cardiovascular risk**	**Blood pressure (mmHg)**
TIPS 3 - (16)	1- Polycap (thiazide 25 mg, atenolol 100 mg, ramipril 10 mg, simvastatin 40 mg) taken once daily 2- Aspirin 75 mg daily 3- Vitamin D 60,000 IU monthly	Matching placebo	5,713		63.9 (6.6)		—	SBP: 144.5 (16.8) DBP: 83.9 (9.7)
HOPE 3	1- polypill (Candesartan 16 mg/HCT 12.5 mg) 2- Rosuvastatin 10 mg	Placebo	(Candesartan /HCTZ) Asian: 3,118 non-asians: 3,238	Asian: 3123 non-asians: 3,226	Asian: 64.75 (5.99) non-asians: 66.40 (6.62)	Asian: 64.88 (6.02) non-asians: 66.41 (6.58)	Framingham risk score, N (%): (polypill) Asian: 23.28 (13.41) non asian: 21.37 (11.99) (placebo) asian: 22.68 (13.31) non-asians: 21.22 (11.96)	(polypill) Asian: 139.86 (14.15)/82.55 (9.14) Non-Asian: 136.61 (15.16) / 81.38 (9.58) (placebo) Asian: 139.82 (14.44) / 82.31 (9.00) Non-Asians: 135.96 (14.9) / 81.21 (9.45)
Polyiran	1- polypill 1, (hydrochloro thiazide 12.5 mg, aspirin 81 mg, atorvastatin 20 mg and enalapril 5 mg.) 2- (those who develop cough at follow up): polypill 2, (valsartan 40 mg instead of enalapril 5 mg)	Minimal care	3,421	3,417	mean (95%CI) 59.3 (59.0–59.6)	59.7 (59.4–60.1)	—	polypill: SBP: 130.2 DBP: 78.5 minimal care: SBP: 131.9 DBP: 79.2
HOPE 4	Polypill: combination of two antihypertensives included an angiotensin receptor blocker or angiotensin converting enzyme inhibitor coupled with a diuretic or calcium channel blocker, with atorvastatin at 20 mg or rosuvastatin at 10 mg	Usual care	644	727	65.1 (9.1)	65.8 (9.7)	Control: 35.5% (22.3) Intervention: 32.6% (21.4)	Control: SBP: 151.7 (15.6) DBP: 85.3 (11.9) intervention: SBP: 152.1 (15.4) DBP: 84.7 (12.0)
SCCS	(polypill) once daily containing: Atorvastatin 10 mg, amlodipine 2.5 mg, losartan 25 mg, and hydrochlorothiazide 12.5 mg.	Usual care	148	155	56 (6)	56 (6)	Control: 13.0 ± 10.1 Intervention: 12.4 ± 8.9	SBP: control: 140 (17) intervention: 140 (18)
Soliman et al. (21)	The polypill (Red Heart pill 2b): -aspirin (75 mg), -simvastatin (20 g), -lisinopril (10 mg) and -hydrochlorothiazide (12.5 mg)	Standard practice (management of their CVD risk according to the usual care given to participants in similar conditions)	105	111	59.0 ± 6.9	59.2 ± 7.4	Polypill: 44.1 ± 20.3 standard practice: 41.6 ± 19.8	SBP: 165.2 ± 18.2
Malekzadeh	Polypill: • Aspirin (81 mg) • Enalapril (2.5 mg) • Etorvastatin (20 mg) • Hydrochlorothiazide (12.5 mg)	Placebo	241	234	59.0 ± 6.5	59.1 ± 7.3	—	Polypill: SBP: 124.8 ± 17.3 DBP: 78.4 ± 10.4 placebo: SBP: 130.3 ± 17.4 DBP: 81.2 ± 9.7
OLSTA	1- FDC therapy (40 mg olmesartan medoxomil, 20 mg rosuvastatin) 2- 40 mg olmesartan medoxomil 3- 20 mg rosuvastatin	Placebo	1-FDC therapy group = 61 2-Olmesartan medoxomil group = 36 3-Rosuvastatin group = 36	29	1-FDC therapy group = 61.9 (8.1) 2-Olmesartan medoxomil group = 59.5 (6.9) 3-Rosuvastatin group = 61.8 (8.0)	62.5 (8.2)		-Intervention: 1-FDC therapy group = SBP: 150.6 (11.9) DBP: 92.0 (7.4) 2-Olmesartan medoxomil group = SBP: 150.6 (15.5) DBP: 93.3 (5.0) 3-Rosuvastatin group = SBP: 148.9 (13.3) DBP: 92.9 (6.5) -Control: SBP: 152.2 (14.5) DBP: 92.5 (7.0)

**Figure 3:**
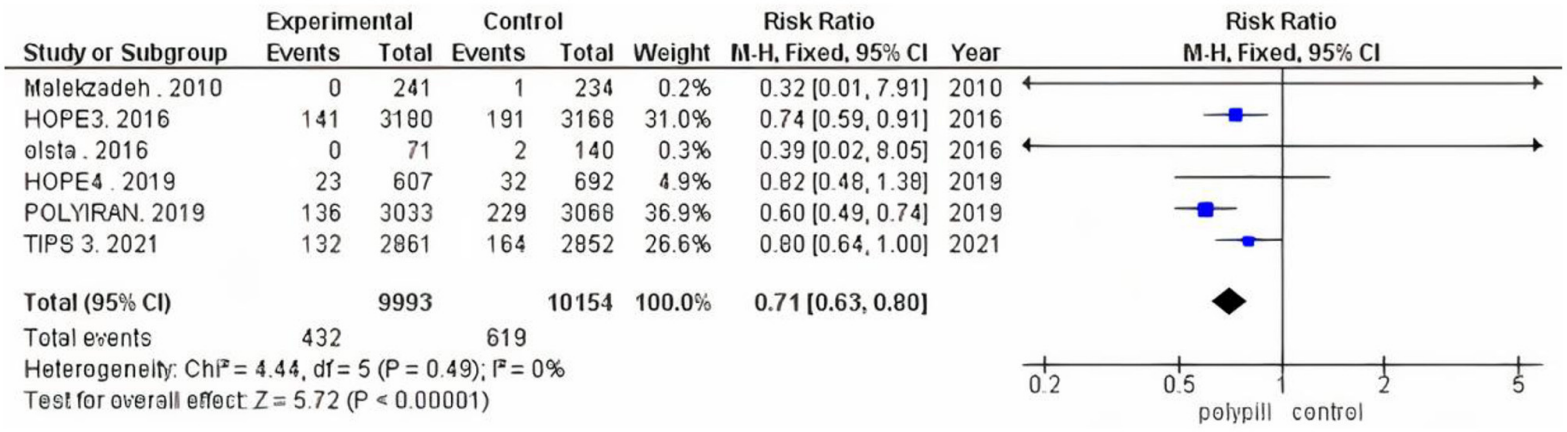
Forest plot of total fatal and non-fatal CV events.

The total number of patients in the six included clinical trials with the outcome of fatal and non-fatal cardiovascular events is 20,147. Of the 9,993 who received a polypill, 432 experienced a fatal or non-fatal cardiovascular event, as compared to 619 out of 10,154 in the placebo/minimal care group. The pooled risk ratio for patients in the polypill treatment group was 0.71 (95% CI 0.63 to 0.80, *p* value > 0.00001), as compared to patients in the placebos/minimal care group. No publication bias was observed. We also found no statistically significant heterogeneity among the included studies (*p* = 0.49), as shown in Figure 3.

**Figure 4:**
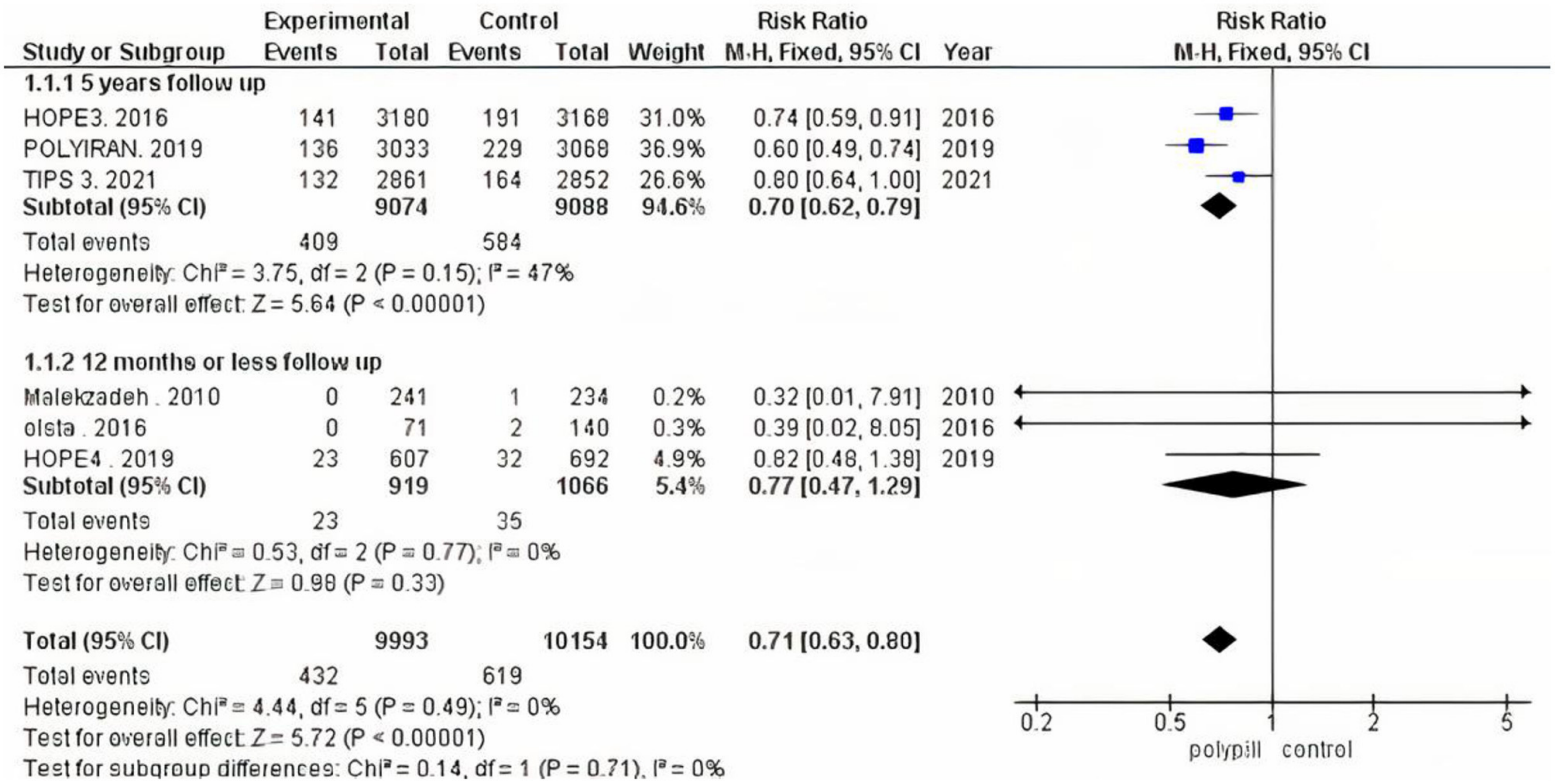
Forest plot of the 12-months-or-less and 5-year follow-up subgroup analysis.

We performed subgroup analysis because trials varied according to their follow-up duration. We ran one analysis for the three trials that had a follow-up period of 12 months or less, and another analysis for the three that reported a 5-year follow-up period. The pooled risk ratio of the 12-months-or-less follow-up subgroup between patients who received the polypill treatment and patients who received minimal care was 0.77 (95% CI 0.47 to 1.29, *p*-value = 0.33). We found no statistically significant heterogeneity among the three studies (*p* = 0.77), as shown in Figure 4. The pooled risk ratio of the 5-year follow-up subgroup between patients who received the polypill treatment and patients who received placebos was 0.70 (95% CI 0.62 to 0.79, *p*-value < 0.00001). No statistically significant heterogeneity was found among the studies (*p* = 0.15), as shown in Figure 4.

**Figure 5:**
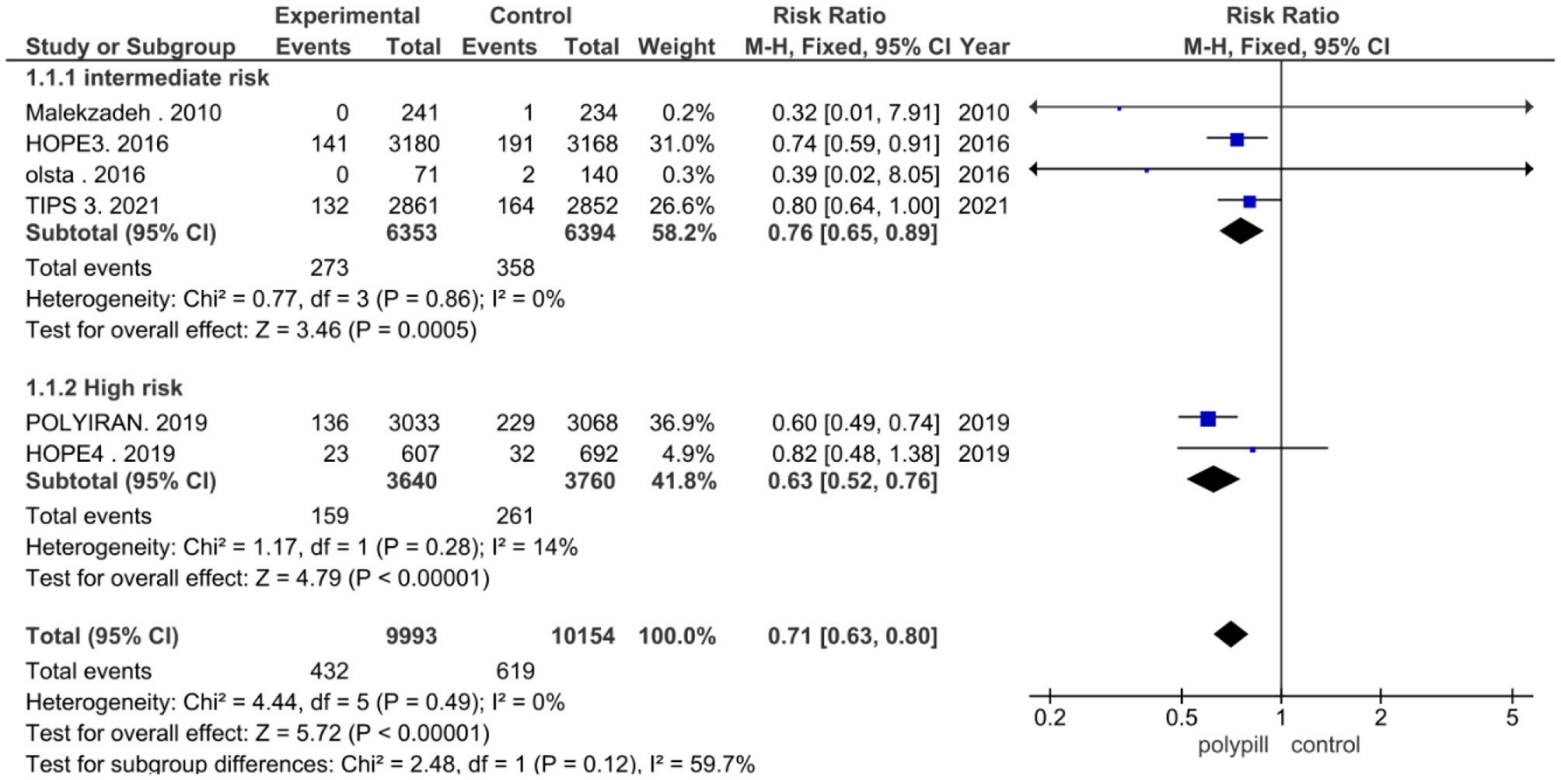
Forest plot according to risk stratification: intermediate and high-risk patients subgroup analysis.

We performed a third subgroup analysis, as four clinical trials studied patients with an intermediate risk of cardiovascular disease, while two trials studied patients with a high risk of cardiovascular disease. The pooled risk ratio between patients who received the polypill treatment and patients who received placebos/minimal care in the intermediate-risk subgroup was 0.76 (95% CI 0.65 to 0.89, *p* value = 0.0005). We found no statistically significant heterogeneity among the four studies (*p* = 0.86), as shown in Figure 5. The pooled risk ratio between patients who received the polypill treatment and patients who received placebos/minimal care in the high-risk subgroup was 0.63 (95% CI 0.52 to 0.76, *p*-value > 0.00001). No statistically significant heterogeneity was found between the studies (*p* = 0.28), as shown in Figure 5.

**Figure 6:**
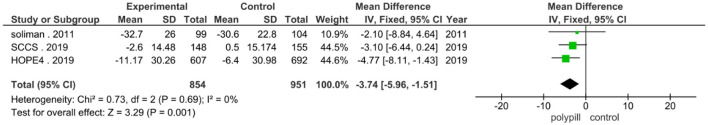
Forest plot of the difference in the 10-year predicted cardiovascular risk.

The total number of patients in the three trials that were analyzed for the difference in 10-year predicted cardiovascular disease risk is 1,805 (854 received the polypill treatment and 951 received placebos/minimal care). The pooled mean difference for patients in the polypill group was −3.74 (95% CI −5.96 to −1.51, *p*-value = 0.001), as compared to those in the minimal-care group. No statistically significant heterogeneity was found among the three included studies (*p* = 0.69), as shown in Figure 6.

### Safety

**Figure 7:**
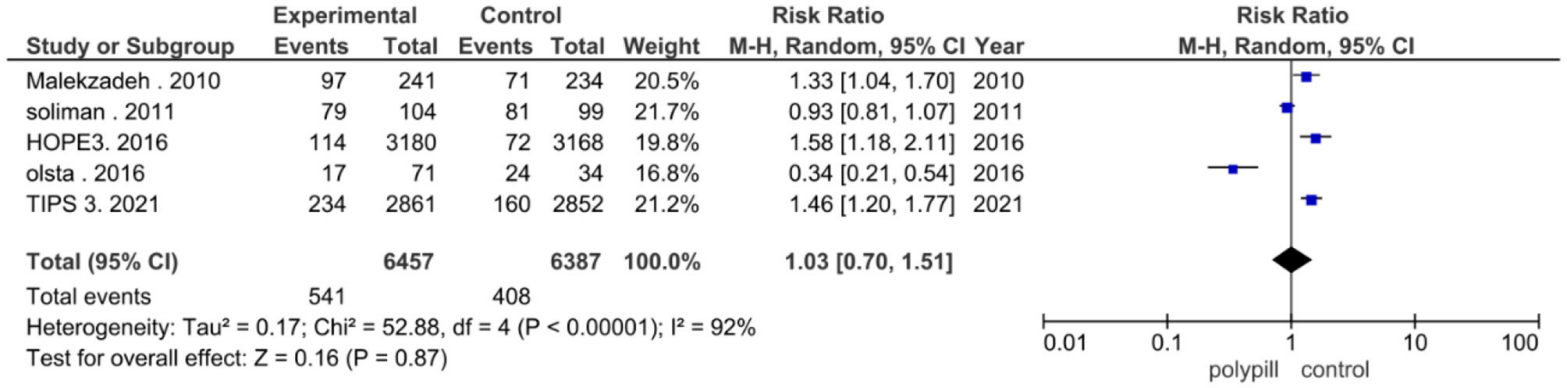
Forest plot of the total adverse events.

Five studies reported the total number of adverse events experienced by patients. Five hundred and forty one out of a total of 6,457 patients in the polypill group experienced adverse events, compared to 408 out of 6,387 patients in the minimal care/placebo group. The pooled risk ratio of total adverse events between patients who received the polypill treatment and patients who received minimal care/placebo was 1.03 (95% CI 0.70 to 1.51, *p*-value = 0.87). Statistically significant heterogeneity was found among the five studies, which was not resolved by using leave-one-out test (*p* < 0.00001), as shown in Figure 7.

**Figure 8:**
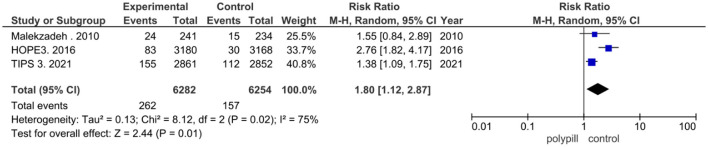
Forest plot of treatment discontinuation due to adverse events.

Discontinuation of treatment due to adverse events was reported in three of the eight clinical trials that were included in our meta-analysis. Two hundred and sixty two out of the 6,282 patients in the polypill group of these trials discontinued the polypill due to adverse events, while 157 in the placebo/minimal care group discontinued treatment due to adverse events. The pooled risk ratio of discontinuation due to adverse events between patients who received the polypill treatment and patients who received minimal care/placebo was 1.80 (95% CI 1.12 to 2.87, *p*-value = 0.01). We found statistically significant heterogeneity among the three studies), as shown in Figure 8, so we performed leave-one-out test by removing (26) study, and the heterogeneity was solved (*p* = 0.73) and the results were (RR = 1.40, [95% CI = 1.12 to 1.75], *P* = 0.003).

**Figure 9:**
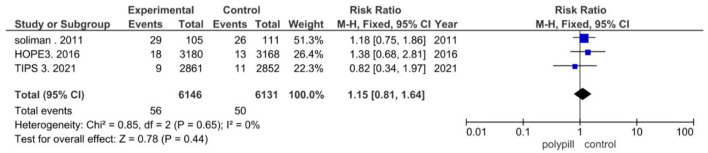
Forest plot of myalgia.

In the included clinical trials, myalgia was found to be the most reported adverse event. Three of the eight trials in our meta-analysis reported myalgia. Of the 6,146 patients in the polypill group of these three trials, myalgia was reported in 56 patients; of the 6,131 patients in the minimal care/placebo group, myalgia was reported in 50 patients. The pooled risk ratio of myalgia between patients who received the polypill treatment and patients who received minimal care/placebo was 1.15 (95% CI 0.81 to 1.64, *p*-value = 0.44). We found no statistically significant heterogeneity among the three trials (*p* = 0.65), as shown in Figure 9.

## Discussion

Our meta-analysis suggests that a polypill regimen for primary prevention in patients with intermediate- to high- cardiovascular risk reduces the incidence of fatal and non-fatal cardiovascular outcomes, including death from cardiovascular causes, myocardial infarction, stroke, heart failure, resuscitated cardiac arrest, arterial revascularization, and angina. Eight studies met inclusion criteria, wherein the pooled outcomes of 10,240 patients receiving a polypill were compared with 10,413 patients who did not receive a polypill. Previous studies revealed that the effect of the polypill on fatal and non-fatal ASCVD events was uncertain (8). This may have been due to low-quality evidence, since previous trials had a short duration and were not designed to assess the clinical outcome of the polypill (rather, the data used in analysis were mainly reported as adverse events). The previous trials had several disadvantages that were overcome in the trials included in our meta-analysis, and results could not be generalized because they were restricted to low income and non-developed countries. In our analysis, the HOPE 3 study included 21 countries throughout the world, such as the United States, Australia, and multiple countries in Europe. The TIPS 3 study also included patients from North America. Another major pitfall of previous trials was that they often reported the effect of the polypill on the CV risks themselves, such as blood pressure and blood lipid levels, rather than clinical outcomes. Again, this issue was resolved in our meta-analysis by several trials that were specifically designed to assess the effect of the polypill on a composite of CV deaths, MI, stroke, and revascularisation. In these studies, such as TIPS 3, HOPE 3, and PolyIran, we witnessed a pooled risk ratio between the polypill and comparator groups of 0.70 (95% CI 0.62 to 0.79, *p*-value > 0.00001). In studies that did not report these outcomes, we instead analyzed the 10-year predicted cardiovascular risk – for example, the SCCS and Soliman trials showed a significant −3.74 (95% CI −5.96 to −1.51, *p*-value = 0.001) mean difference between treatment and minimal care/placebo groups.

Interestingly, the efficacy of the polypill was witnessed in a phase 4 study performed on 1,193 patients in Mexico – the treatment showed even better-than-expected improvement of all-cause mortality and vascular-related mortality, as compared to a phase 3 trial, after the use of the CNIC-Ferrer polypill (22). Lastly, the problem of short trial duration – rendering the evidence of previous studies uncertain – was corrected by multiple trials in our analysis having a mean follow up of 5 years. Apart from the trial design of previous studies, the polypill treatment itself was flawed in multiple ways, with the inability to tailor dosage and individualize therapy as the most pressing issue. However, a range of different formulations and doses – as opposed to only one – might surmount this problem. Patients could potentially be stratified based on their risk scores, with a different formulation and different dose used in each stratum. Additionally, as was done in the TIPS 3 study, any patient who might still have uncontrolled blood pressure or dyslipidaemia could be prescribed additional medication. Another barrier of previous research was the agreement of patients to use a polypill while being asymptomatic, as well as their fear of adverse events while using pharmaceutically active components. However, an interview surveying Australian patients showed that they favored using a prophylactic approach (23). Our meta-analysis showed no significant difference in the total adverse events between the polypill and the comparator group (*p* = 87), which suggests that the polypill is safe to use as a preventive measure over long periods of time. The willingness of physicians to prescribe polypills to their patients was also considered, as many seemed reluctant about the risk-benefit ratio of such a treatment. This meta-analysis may therefore serve as evidence to convince hesitant physicians about the benefits of these fixed-dose combination drugs. The advantages of a polypill regimen, as proven by our meta-analysis, include a decrease in the incidence of fatal and non-fatal CV events. In addition, polypill treatment is cost effective (24, 25); however, cost reductions may still be required in some developing countries (26).

There are many areas of interest that require further study regarding polypill use in primary prevention for intermediate- and high- cardiovascular risk patients. Firstly, additional research is needed in order to determine the most convenient drug combination possible for the reduction of the incidence of ASCVD events. Secondly, polypill trials that stratify patients according to their cardiovascular risk score should be designed and conducted.

### Implication for Future Practice

Evidence has shown that a risk-based strategy is better than a blood pressure-based approach or a combination (blood pressure and risk) strategy in terms of cardiovascular disease prevention (9). For that reason, we suggest that the prescription and use of a polypill be based upon risk scores, as shown in studies such as the TIPS 3, HOPE 4, and SCCS trials. Efficient CVD prevention should include the judicious use of evidence-based protocols, founded in the practices of risk-based management and a team approach. Strategies to reduce CVD should integrate socioenvironmental approaches and community resources into physician care, as well. This multidimensional treatment plan was successfully illustrated in the HOPE 4 study, which proved that a comprehensive model of care involving physicians and family substantially improved blood pressure control and reduced cardiovascular disease risk (13, 27).

### Limitations of This Meta-Analysis

Due to the lack of clinical outcomes in some studies (such as the SCCS and Soliman trials), we had to utilize the difference in 10-year predicted cardiovascular risk as another assessment tool in our analysis.

## Conclusion

A polypill that combines a lipid-lowering and blood pressure-lowering drug reduces the incidence of fatal and non-fatal cardiovascular events in patients with an intermediate and high risk of cardiovascular disease and could be used as a primary preventive approach in these patients. Limitations of previous studies regarding the polypill were all corrected by the results of the new trials that were included in this meta-analysis. The fear that the polypill may be a scattergun approach in primary prevention, leaving a rather asymptomatic population sentenced to lifelong treatment, was disproven by our study.

## Data Availability Statement

The original contributions presented in the study are included in the article/supplementary material, further inquiries can be directed to the corresponding author/s.

## Author Contributions

OK: design and concept and writing and review. KM: data analysis and interpretation. MA: data extraction. JS: review and editing. All authors contributed to the article and approved the submitted version.

## Conflict of Interest

The authors declare that the research was conducted in the absence of any commercial or financial relationships that could be construed as a potential conflict of interest.

## Publisher's Note

All claims expressed in this article are solely those of the authors and do not necessarily represent those of their affiliated organizations, or those of the publisher, the editors and the reviewers. Any product that may be evaluated in this article, or claim that may be made by its manufacturer, is not guaranteed or endorsed by the publisher.
